# Reframing the End Stage of Renal Disease: How Age Matters

**DOI:** 10.1093/geroni/igaa057.224

**Published:** 2020-12-16

**Authors:** Deborah Waldrop, Patricia Denny, Sandra Lauer, Kathleen Grimm

**Affiliations:** 1 University at Buffalo, Buffalo, New York, United States; 2 Erie County Medical Center, Buffalo, New York, United States

## Abstract

End Stage Renal Disease (ESRD) conveys high symptom burden, multimorbidity and the greater likelihood of hospital death than other serious illnesses. Increases in people with ESRD occurred most sharply among adults age 75+. Despite high mortality risk, few with ESRD consider end-of-life preferences or discuss with a physician. The purpose of this study was to explore the nature of advance care planning in ESRD. The study utilized mixed methods and both qualitative and quantitative data was collected during in-depth chairside interviews with 31 people while they were on hemodialysis. Participants ranged in age from 29-85; Mage=60; (N=13 [40%/)60). The data was divided above and below the Mage, and distinct differences were found in the nature of advanced care planning by age group. Greater numbers of people >60 (N=11[61%]) were not considering a transplant while (N=9[69%]) of those under 60 had a failed transplant and were again on the waiting list. Although the majority of participants had a health care proxy (N=27[87%]), more who were >60 had a proxy who knew their wishes (N=14[78%]) compared with (N=9[69%]) who were 60 compared with none <60. The qualitative data illuminated these age-differentiated responses in themes: Older age and (1) Multimorbidity; (2) Frequency/intensity of hospitalization; (3) Diminished hope of transplantation; and (4) More acute death awareness. The need for disease-specific advance care planning—with hopes and expectations about transplant--and attention to the influence of age and decline cannot be overstated.

